# New method for intraocular lens power calculation using a rotating Scheimpflug camera in eyes with corneal refractive surgery

**DOI:** 10.1038/s41598-020-65827-y

**Published:** 2020-06-02

**Authors:** Kyuyeon Cho, Dong Hui Lim, Young-Sik Yoo, Tae-Young Chung

**Affiliations:** 1 Department of Ophthalmology, Sungkyunkwan University School of Medicine, Samsung Medical Center, Seoul, Korea; 2 Department of Medical Device Management and Research, Samsung Advanced Institute for Health Sciences and Technology, Sungkyunkwan University, Seoul, Republic of Korea; 3 Department of Ophthalmology, College of Medicine, Uijeongbu St. Mary’s Hospital, The Catholic University of Korea, Gyeonggi-do, South Korea

**Keywords:** Outcomes research, Visual system

## Abstract

To introduce and evaluate a refraction-based method for calculating the correct power of the intraocular lens (IOL) in eyes with corneal refractive surgery and to compare the results here to previously published methods. Retrospective review of medical records was done. Group 1 was used to derive two formulas. From the relevant IOL calculation and postoperative refractive data, the refraction-derived K values (Krd) were calculated using a linear regression analysis. The values obtained with the two formulas were compared to previously published methods in group 2 to validate the results. The following methods were evaluated: Haigis-L, Barrett True-K (no history), Potvin-Hill, BESSt 2, Scheimpflug total corneal refractive power (TCRP) 4 mm (Haigis), Scheimpflug total refractive power (TRP) 4 mm (Haigis), modified Scheimpflug TCRP 4 mm (Haigis), and modified Scheimpflug TRP 4 mm (Haigis). The modified TCRP 4 mm Krd (Haigis) had good outcomes, with 60% and 90% of eyes within ±0.50 D and ±1.00 D of the refractive target, respectively. A new method using modified Scheimpflug total corneal refractive power in the 4.0 mm zone appeared to be an accurate method for determining IOL power in eyes with corneal refractive surgery.

## Introduction

As significantly increased number of laser refractive surgery, now it is common to face the patient with cataract after refractive surgery. But unfortunately, it is challenging to satisfy the accuracy of intraocular lens (IOL) calculation of post refractive surgery^1,2^. Mostly the error of the IOL calculation is accompanied by hyperopic surprise in post-myopic laser refractive surgery patients. Various methods have been described to overcome the inaccuracy of post-refractive surgery IOL calculation^3–5^. And currently methods that do not require past clinical data^6–13^ have proven comparable results to traditional clinical history methods.

Groups without the clinical history has popular methods including the Haigis-L^6^, Barrett True-K (no history)^8–10^, Potvin-Hill^11^ and BESSt^13^ they showed improved accuracy than the traditional clinical history methods. These methods do not require historical information but rather indices such as current biometry, keratometer index change, and keratometric correction factor. So, it is very important to accurately measure the actual corneal power after laser refractive surgery for IOL calculation without previous clinical historic data.

Our previous study^14^ reported that formulas using the Scheimpflug total corneal refractive power (TCRP) and the total refractive power (TRP) with the Haigis formula had comparable results to previously published methods (Haigis-L, Shammas, Barrett True-K (no history), and WKM). According to Pentacam rotating Scheimpflug camera system (Oculus Optikgerate GmbH) interpretation guide, TRP measures only the anterior surface of corneal power using standard keratometric index of 1.3375. TNP calculate values of both the anterior and posterior surfaces of cornea. Also TNP use the refractive index of air, cornea, and aqueous humor. TCRP is measured using ray tracing method to calculate the refractive power of the cornea.

The purpose of this study is to introduce and evaluate new refraction-based methods that utilize data measured by Scheimpflug (Pentacam HR; Oculus, Wetzlar, Germany). In addition, by comparing the results seen in our study to previously published methods, we sought to provide new post-refractive IOL calucation methods.

## Methods

Like our previous study^14^, retrospective review was performed on the medical records of a total of 80 patients (80 eyes) who had undergone myopic laser refractive surgery and subsequent cataract surgery at the Samsung Medical Center from January 2010 to December 2018. The study was performed in accordance with the principles of the Declaration of Helsinki and was approved by the Institutional Review Board of the Samsung Medical Center. Institutional Review Board of the Samsung Medical Center waived the requirement of the informed consent for this study.

Consistently with our previous study^14^, the patients had no pre-refractive surgery biometric data and were examined before cataract surgery with a thorough ophthalmologic examination, including slit-lamp examination, visual acuity, manifest refraction, Potential Acuity Meter (P.A.M.), and optical biometry. Optical biometric data (axial length and anterior chamber depth) and corneal power were measured using swept-source OCT (IOL Master 700; Carl Zeiss Meditec, AG). The manifest refraction after cataract surgery was obtained from an examination three months post-surgery. Prediction error (PE) was then calculated by subtracting the predicted refraction from the power of the actual refraction. One surgeon (T.Y.C) performed the cataract surgeries using a temporal clear corneal incision and phacoemulsification. Various methods were used for corneal power estimation and IOL power calculation before surgery. The Surgeon selected the IOL power to be implanted depending on his judgment. Tecnis monofocal (ZCB00) intracocular lenses were implanted in all cases. Optimized IOL constants for the ZEISS IOL Master (User Group for Laser Interference Biometry (ULIB) were used for Haigis and other formulas. Wang L., Koch DD. *et al*.^15^ reported that the keratometric data from swept-source OCT (SS-OCT) biometer (IOLMaster 700, Carl Zeiss Meditec AG) is lens-constant compatible of ULIB and existing formulas and IOL constants can be applied. Furthermore, the keratometric data from IOL Master 700 do not need to be modified and can be used directly in the standard Haigis formula in eyes with corneal refractive surgery^15^.

Haigis-L^6^, Barrett True-K (no history)^8–10^, Potvin-Hill^11^ formulas were calculated using an online calculator provided by the ASCRS(version 4.8 http://iolcalc.ascrs.org/) and the APASCRS (ver 2.0 http://www.apacrs.org/barrett_true_K_universal_2). BESSt^13^ formula was calculated by the BESSt 2 IOL Power Calculatoer (ver.2.0.0.76, http://www.besstformula.com/) software. In addition, K data for cataract surgery IOL calculation were obtained using values from the Scheimpflug system’s TCRP and TRP maps.

Group I was used to find the comparable K values using a Scheimpflug and to derive the equations needed to calculate the refraction derived K (K_rd_). In our previous study, we found that the Scheimpflug TCRP 4 mm K (Haigis) and TRP 4 mm K (Haigis) formulas had comparable accuracies to the Haigis-L^6^, Barret True K (No History)^8–10^, and Potvin-Hill^11^ formulas.

Using the available postoperative refractive data for each patient in group I, the “refraction-derived K (K_rd_)” using the actual refraction data after cataract surgery was back -calculated with IOL Master 700 as the K value that would have minimized the difference between the target refraction and the actual refraction. They involved a back calculation based on the IOL in the eye, the K values, and the target refraction. To establish the regression formula, the measured K values and the calculated K_rd_ values were plotted on a scattergram, and the best-fit regression equation was obtained. We used the Pearson product-moment correlation coefficient to evaluate each scattergram correlation. We also calculated the average K values obtained with each formula.

A second group that included 30 eyes (Group II) was then evaluated for validation of the derived formulas. In each case, we calculated K_rd_ using the regression formula and compared the results of the IOL calculation from the Haigis-L, Barrett True-K (no history), Potvin-Hill, BESSt, Scheimpflug TCRP 4 mm (Haigis), and Scheimpflug TRP 4 mm (Haigis) methods. Mean numerical error, mean absolute error, and percentage of eyes within a refractive PE of ± 0.5 diopters (D) and ± 1.0 D were calculated for each method.

The data was in normal distribution, so 1-sample t test was used to evaluate the mean numerical PE were significantly different from zero. Analysis of variance was used to compare differences in numerical PE between methods by using the F-test. The Friedman test was usedto compare the mean absolute PE using different formulas. Percentage of eyes within 0.5 D and 1.0 D of the target refraction were compare between the formulas using the McNemar test. If a significant difference was obtainedbetween formulas, the post hoc analysis with the Wilcoxontest or McNemar test was used for pairwise comparisons. The Bonferroni correction was applied for multiple tests. Statistical analysis was performed using Microsoft Excel 2007(Microsoft Corp., Redmond, WA) and SPSS version 22 (SPSS Inc., Chicago, IL). Statistical significance was defined as *p* < 0.05.

## Results

**Figure 1:**
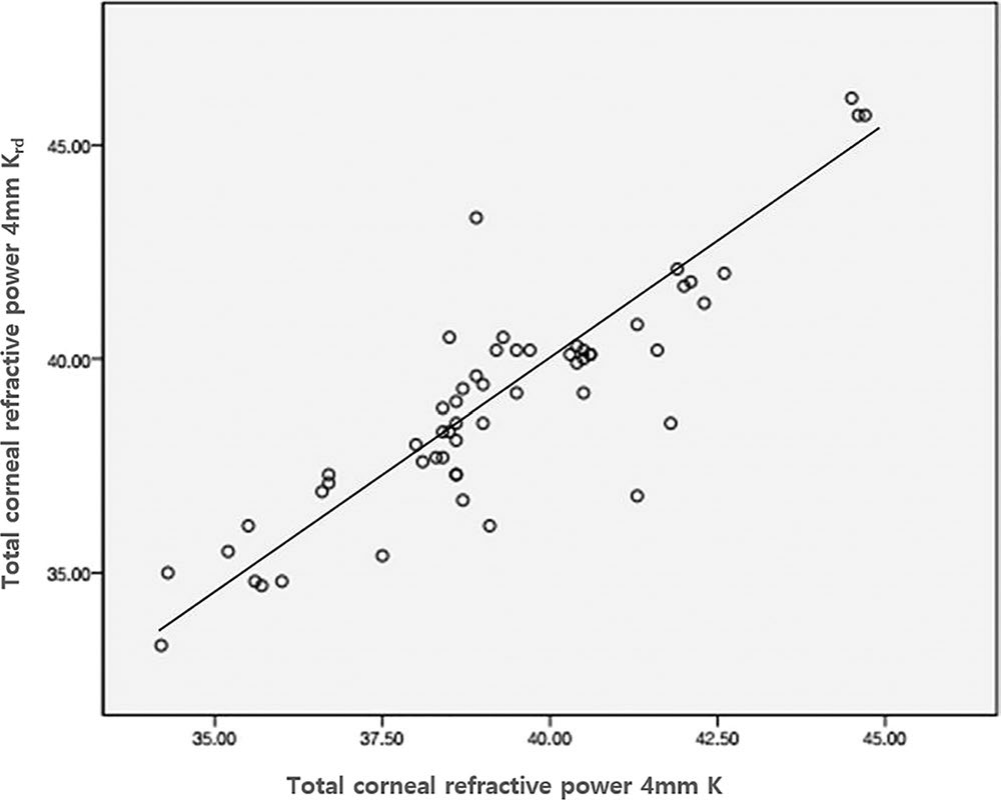
Scattergram of the Scheimpflug measured values (Diopters (D)) TCRP 4.0 mm zone (K) versus calculated values using the refraction-derived method (K_rd_) in Group I.

**Figure 2:**
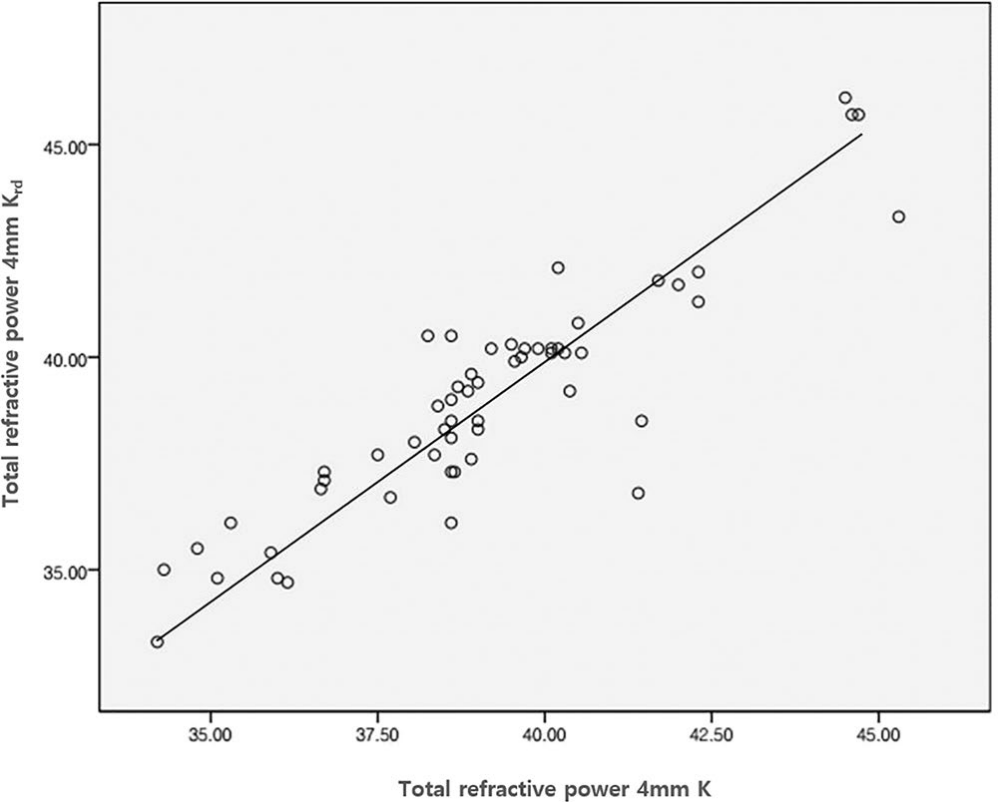
Scattergram of the Scheimpflug-measured values (Diopters (D)) TRP 4.0 mm zone (K) versus the calculated values using the refraction-derived method (K_rd_) in Group I.

A total of 80 patients (80 eyes) who had undergone myopic laser refractive surgery and subsequent cataract surgery were included in this study. Tables 1 and 2 show the average values, standard deviation, and the data range for the two study groups. To establish a formula, the measured values ofScheimpflug TCRP 4.0 mm zone K (K_TCRP_), K_TRP,_back-calculated K_rd.TCRP_, and back-calculated K_rd.TRP_ were plotted on a scattergram (Fig. 1. And Fig. 2.). The best fit regression equation was Y = 0.989x + 0.179 and Y = 0.974x+ 0.882 with correlation coefficientsof 0.873 (*p* < 0.001) and 0.898 (*p* < 0.001), respectively. Using this equation and based only on the measured K values (K_TCRP_ and K_TRP_), corrected K values were then calculated usinga refraction-derived method, where

**Table 1:** Preoperative patient demographics of the 50 patients (50 eyes) in group 1 and 30 patients (30 eyes) in Group 2 acquired before cataract surgery.

	Group 1	Group 2	*p*-value
Age, mean ± SD	54.64m± 9.37 (38–76)	55.4 ± 6.65 (43–70)	0.351
Eye, OD/OS	27/23	17/13	
Sex, M/F	21/29	10/20	
UCVA (logMAR)	0.64A± 0.49 (0.15–1)	0.63 ± 0.28 (0.15–1)	0.483
BCVA (logMAR)	0.34A± 0.23 (0–1)	0.38 ± 0.33 (0–1)	0.521
Spherical Equivalent	−2.83r± 3.52 D (−5.25–0.75 D)	−2.90 ± 2.42 D (−5.25 – −0.25 D)	0.637
Biometry			
Axial Length (PCI)	28.08 ± 2.50 mm (22.55–31.62)	28.212± 2.24 mm (24.03–31.25)	0.191
ACD (PCI)	3.63(± 0.34 mm (2.83–4.3)	3.612± 0.32 mm (3.17–4.49)	0.386
Mean K (PCI)	39.73K± 2.23 D (34.45–45.96)	38.826± 2.94 D (37.38–45.96)	0.415

UCVA: uncorrected visual acuity, BCVA: best-corrected visual acuity, PCI: partial coherence interferometry, ACD: anterior chamber depth, K: keratometry.

**Table 2:** A comparison of mean numeric and mean absolute prediction errors (PE) between previously known formulas.

	Numeric PE	Variances (D^2^)	Absolute PE^**^	Variances (D^2^)	± 0.5 D ^(%)^	± 1.0 D ^(%)^
Haigis-L	0.15 ± 0.79	0.62	0.63 ± 0.47	0.22	53.3%	83.3%
Barrett True-K	0.19 ± 0.74	0.55	0.52 ± 0.76	0.58	46.7%	80%
Potvin-Hill	0.06 ± 0.83	0.69	0.45 ± 0.55	0.30	50%	83.3%
BESSt 2	0.26 ± 0.78	0.64	0.5 ± 0.63	0.40	50%	83.3%
TCRP 4 mm K	0.21 ± 0.87	0.75	0.59 ± 0.53	0.75	46.7%	76.7%
TCRP 4 mm Krd	0.20 ± 0.65	0.42	0.46 ± 0.40	0.28	60%	90%
TRP 4 mm K	−0.06 ± 1.08	1.18	0.75 ± 0.69	0.48	50%	73.3%
TRP 4 mm Krd	0.00 ± 0.74	0.55	0.54 ± 0.66	0.44	53.3%	83.3%

$${\bf{K}}{\bf{r}}{\bf{d}}{\boldsymbol{.}}{\bf{T}}{\bf{C}}{\bf{R}}{\bf{P}}\,{\boldsymbol{=}}{\bf{0.989}}{\bf{K}}{\bf{T}}{\bf{C}}{\bf{R}}{\bf{P}}{\boldsymbol{+}}{\bf{0.179}}\,{\bf{a}}{\bf{n}}{\bf{d}}$$

$${\bf{K}}{\bf{r}}{\bf{d}}{\boldsymbol{.}}{\bf{T}}{\bf{R}}{\bf{P}}\,{\boldsymbol{=}}{\bf{0.974}}{\bf{K}}{\bf{T}}{\bf{R}}{\bf{P}}\,{\boldsymbol{+}}{\bf{0.882.}}$$

The average values of K_TCRP_ and K_TRP_ were 39.24 ± 2.38 D and 39.13 ± 2.49 D, respectively, whereas the average values of K_rd_._TCRP_ and K_rd_._TRP_ were 38.74 ± 2.67 D and 38.87 ± 2.63 D, respectively.

### Numeric and absolute prediction errors

**Figure 3:**
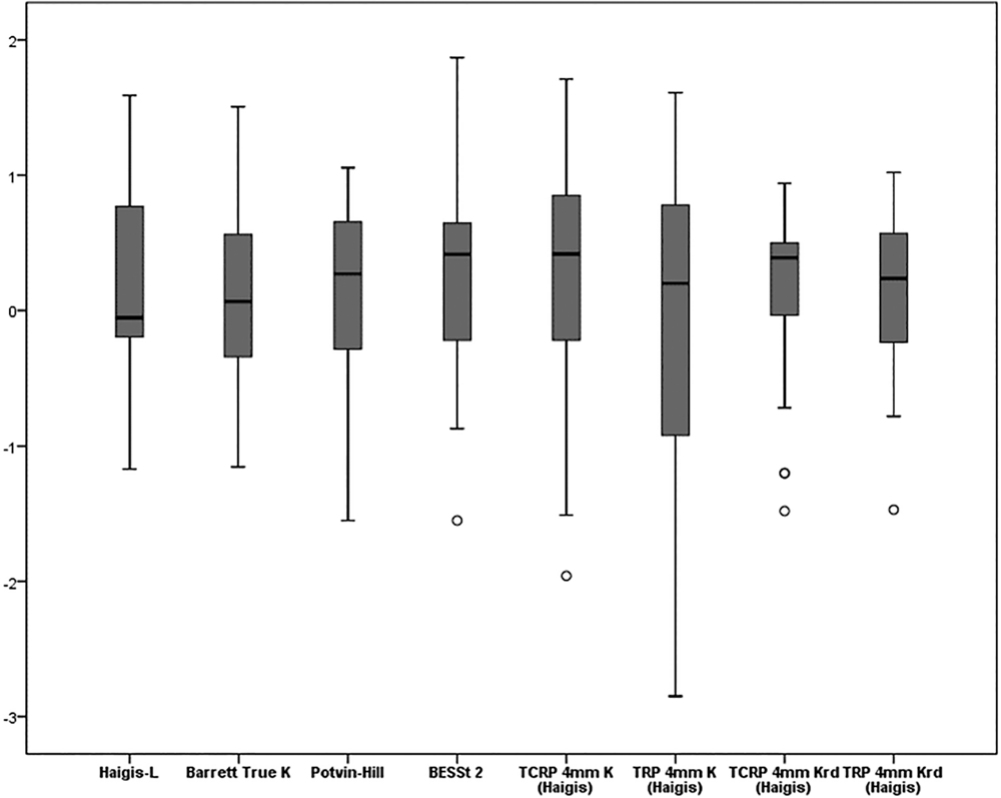
Mean numeric prediction errors (Diopters (D)). A comparison of mean arithmetic PEs between previously known formulas (Haigis-L, Barrett True-K, Potvin-Hill and BESSt 2) and Scheimpflug methods [TCRP 4 mm K (Haigis), TRP 4 mm K (Haigis), TCRP 4 mm Krd (Haigis), and TRP 4 mm Krd (Haigis)]. TCRP: total corneal refractive power, TRP: total refractive power. The bands inside the boxes represent the mean values of numeric and absolute prediction errors. And the each ends of the whiskers represents the maximum and minimum values.

**Figure 4:**
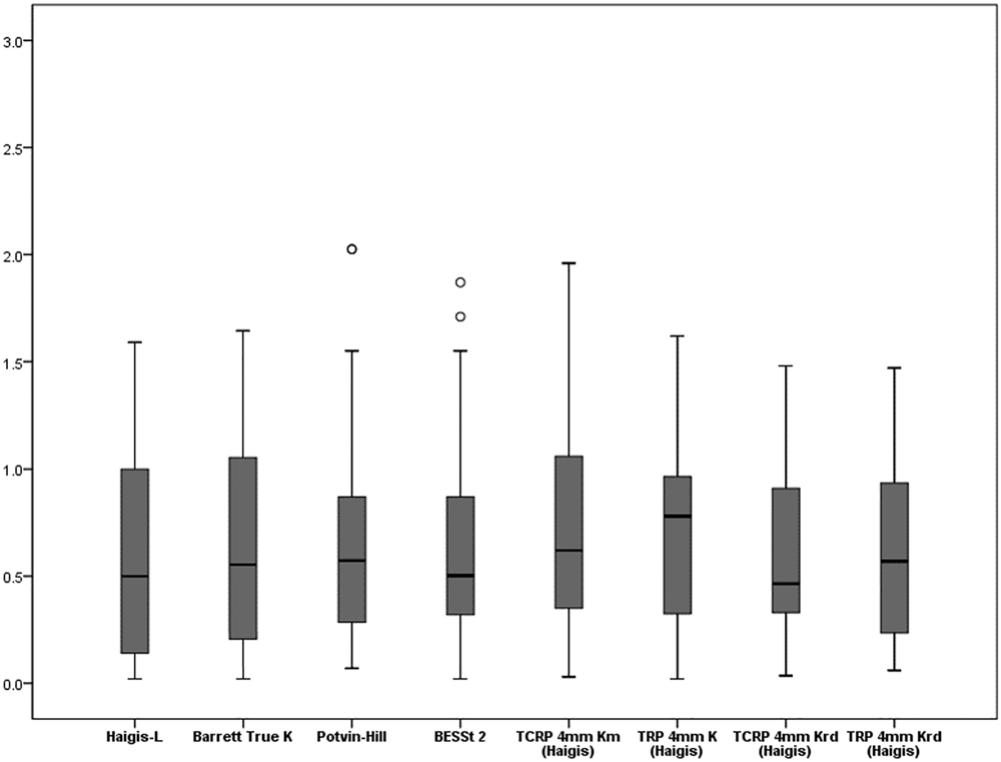
Mean absolute prediction errors. (Diopters (D)). A comparison of mean absolute PEs between previously known formulas (Haigis-L, Barrett True-K, Potvin-Hill and BESSt 2) and Scheimpflug methods [TCRP 4 mm K (Haigis), TRP 4 mm K (Haigis), TCRP 4 mm Krd (Haigis), and TRP 4 mm Krd (Haigis)]. TCRP: total corneal refractive power, TRP: total refractive power. The bands inside the boxes represent the mean values of numeric and absolute prediction errors. And the each ends of the whiskers represents the maximum and minimum values.

The mean numeric PEs of the formulas ranged from −0.06 to 0.41D. Variances of numeric PE of the formulas ranged 0.42 D2 to 1.18 D2. The variances of the TCRP 4 mm Krd (Haigis) was significantly lower than that of all other previously known methods (Haigis-L, Barrett True-K (no history), Potvin-Hill, BESSt) and TCRP 4 mm K (Haigis) (all *p* < 0.001). (Fig. 3, Table 2). Median Absolute PEs of the formulas ranged 0.46 to 0.78D. The TCRP 4 mm Krd (Haigis) median absolute PE was significantly lower than the median absolute PE of all other previously known methods (Haigis-L, Barrett True-K (no history), Potvin-Hill, BESSt) and TCRP 4 mm K (Haigis) (all *p* < 0.001) (Fig. 4, Table 2). Both variances of numeric PE and median absolute PE were not significantly different between the TCRP 4 mm Krd (Haigis) and TRP 4 mm Krd (Haigis).

### Percentages relative to target refraction

**Figure 5:**
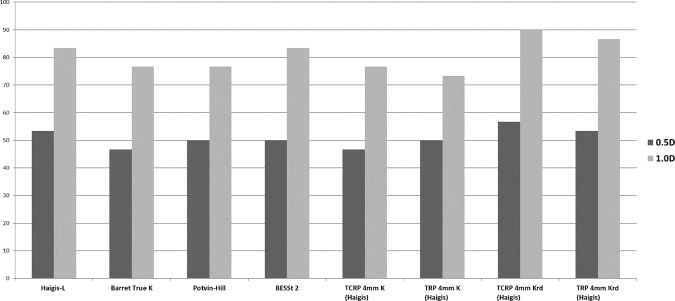
Percentages of eyes within ± 0.5 Diopters (D) and ± 1.0 D of the target refraction. A comparison of percentages of eyes within ± 0.5 D and ± 1.0 D of the target refraction with previously known formulas (Haigis-L, Barrett True-K, Potvin-Hill and BESSt 2) and Scheimpflug methods [TCRP 4 mm K (Haigis), TRP 4 mm K (Haigis), TCRP 4 mm Krd (Haigis), and TRP 4 mm Krd (Haigis)]. TCRP: total corneal refractive power, TRP: total refractive power.

The percentages of each formula combination with predictions between ±0.5 D and ±1.0 D of the target refraction were also evaluated. The percentage of eyes with a prediction error within ±0.5 D of target refraction was significantly higher with the TCRP 4 mm Krd (Haigis) formula than with the all other previously known methods (Haigis-L, Barrett True-K (no history), Potvin-Hill, BESSt) and TCRP 4 mm K (Haigis) (all *p* < 0.001). Also, the percentage of eyes with a refractive prediction error within ±1.00 D of target refraction was significantly higher with the TCRP 4 mm Krd (Haigis) formula than with the all other previously known methods (Haigis-L, Barrett True-K (no history), Potvin-Hill, BESSt) and TCRP 4 mm K (Haigis) (all *p* < 0.001). (Fig. 5, Table 2). Both percentage of eyes with a prediction error within ±0.5 D and ±1.00 D of target refraction were not significantly different between the TCRP 4 mm Krd (Haigis) and TRP 4 mm Krd (Haigis).

## Discussion

By utilizing the Pentacam system to calculate IOL in patients with previous myopic refractive surgery,our aim was to find the most accurate corneal power. Among the numerous corneal power values (TCRP, TRP, and TNP) in the Pentacam system, our previous study^14^ showed comparable results between TCRP and TRP 4 mm K with the Haigis formula.

To improve accuracy, corneal power using the Pentacam system required significant modification including use of the Shammas^7^ no history formula. Additional regression analysis was performed to adjust the K values of TCRP and TRP.

Both new formulas [TCRP 4 mm K_rd_ (Haigis) and TRP 4 mm K_rd_ (Haigis)] using refraction-derived K values were more accurate than the original formulas [TCRP 4 mm K (Haigis) and TRP 4 mm K (Haigis)] using the Scheimpflug measured K values. In addition,the British National Health Service (NHS) has proposed two benchmark standards: 55% and 85% within ± 0.5 D and ± 1.0 D, respectively, of the targeted SE for refractive outcomes following cataract surgery in normal eyes^16^. In our study, only TCRP 4 mm K_rd_ (Haigis) satisfied the NHS benchmark, even in eyes with corneal refractive surgery.

Many previous reports have suggested true net power for providing successful outcomes for post-myopic LASIK eyes^11,17^. In particular, Potvin *et al*.^11^ reported a new Potvin-Hill formula using TNP, which resulted in 34%, 66%, and 91% of eyes within ±0.25 D, ± 0.50 D, and ±1.00 D of the refractive target, respectively. However in our previous study^14^, TCRP with the Haigis formula was more accurate than TNP with the Haigis formula.. Furthermore, our results showed TCRP 4 mm K_rd_ (Haigis) was more accurate than the Potvin-Hill Pentacam formula with outcomes of 90% and 83.3% of eyes within ±1.00 D of the refractive target, respectively.

The results of TCRP 4 mm K_rd_ (Haigis) were more accurate than previously published methods (Haigis-L, Barrett True K, Potvin-Hill and BESSt), with the promising outcomes of mean absolute error 0.46 D and 60% within the ±0.5 D and 90% within the ±1.0 D of the refractive PE, respectively. In addition, the TRP 4 mm K_rd_ (Haigis) formula generated the most emmetropic results among the formulas, along with the least mean numeric error (0.00 ± 0.74 D).

Our study showed the result in the percentage of eyes within ±1.00 D of the target refraction 83.3%, 80% in Haigis-L, Barrett True-K (No History) respectively. It was lower than those of the reported in studies by Potvin - Hill^11^ and Schuster *et al*.^18^ But in contrast, showed better results than those in studies by Abulafia *et al*.^10^, Yang R *et al*.^19^ and McCarthy M. *et al*.^4^ Recently, Wong^20^ reported that the Haigis-L formula was less accurate in Asian than Caucasian populations. In this study, 62 eyes were analyzed with a mean axial length of 27.70 ± 1.53 mm, the absolute error was 0.87 ± 0.62 D and the percentage of correct refractive predictions within ±1.00D was 60.5%. Wong stated that the result of these discrepancies was probably related to the differences in population groups. So particularly in Asian populations, we expect TCRP and TRP 4 mm Krd (Haigis) to be promising alternatives to Haigis-L and the other formulas.

Our results showed that both new formulas [TCRP 4 mm K_rd_ (Haigis) and TRP 4 mm K_rd_ (Haigis)] were comparable to previously known methods. Also, TCRP 4 mm K_rd_ (Haigis) were better than the original TCRP 4 mm K (Haigis). In case between two new formulas [TCRP 4 mm K_rd_ (Haigis) and TRP 4 mm K_rd_ (Haigis)], the results showed no significant difference of the TCRP 4 mm K_rd_ (Haigis) and TRP 4 mm K_rd_ (Haigis).

The fact that total corneal refractive power was the most accurate K value in the Pentacam system is obvious. After refractive surgery, the relative curvatures of the anterior and posterior corneal surfaces are significantly changed. Thus, traditional ratios cannot be applied. Total corneal refractive power accurately measures both anterior and posterior corneal curvatures instead of applying ratio assumptions between the two surfaces. Furthermore, simple regression analysis generated refraction-derived K values that produced better IOL power calculations than those from previously known formulas.

A limitation of this study include: 1) All patients had myopic correction. To use this method universally in comparisons of the accuracy of a modified TCRP (Haigis) after a refractive procedure, further study is needed on its application to eyes with hyperopic refractive correction with an accurate IOL calculation formula. 2) The ULIB optimized lens constants were used for IOL power calculation. Mostly in clinics, surgeons tends to use ULIB lens constants instead of personalized lens constants for eyes with previous corneal refractive surgery^15^.

In conclusion, we introduced a new refraction-derived method to calculate correct corneal power. Furthermore, TCRP and TRP 4 mm K_rd_ (Haigis) showed promise as a means to calculate IOL power in eyes with refractive surgery and where the historical data are absent and as an alternative to previously published methods (Hagis-L and Barrett True K). In addition, TCRP and TRP 4 mm K_rd_ (Haigis) could be better alternatives, especially in an Asian population. Based on our data, we recommend implementing TCRP 4 mm K_rd_ for true corneal power and thus, reliable IOL power calculations.
